# Superinsulating BNNS/PVA Composite Aerogels with High Solar Reflectance for Energy-Efficient Buildings

**DOI:** 10.1007/s40820-022-00797-6

**Published:** 2022-02-02

**Authors:** Jie Yang, Kit-Ying Chan, Harun Venkatesan, Eunyoung Kim, Miracle Hope Adegun, Jeng-Hun Lee, Xi Shen, Jang‐Kyo Kim

**Affiliations:** 1 Department of Mechanical and Aerospace Engineering, The Hong Kong University of Science and Technology, Clear Water Bay, Kowloon, Hong Kong People’s Republic of China; 2 Department of Aeronautical and Aviation Engineering, The Hong Kong Polytechnic University, Hung Hom, Kowloon, Hong Kong People’s Republic of China

**Keywords:** Boron nitride nanosheets, Solvent-assisted freeze-casting, Thermally insulating aerogel, Solar reflectance, Energy-saving buildings

## Abstract

With the mandate of worldwide carbon neutralization, pursuing comfortable living environment while consuming less energy is an enticing and unavoidable choice. Novel composite aerogels with super thermal insulation and high sunlight reflection are developed for energy-efficient buildings. A solvent-assisted freeze-casting strategy is used to produce boron nitride nanosheet/polyvinyl alcohol (BNNS/PVA) composite aerogels with a tailored alignment channel structure. The effects of acetone and BNNS fillers on microstructures and multifunctional properties of aerogels are investigated. The acetone in the PVA suspension enlarges the cell walls to suppress the shrinkage, giving rise to a lower density and a higher porosity, accompanied with much diminished heat conduction throughout the whole product. The addition of BNNS fillers creates whiskers in place of disconnected transverse ligaments between adjacent cell walls, further ameliorating the thermal insulation transverse to the cell wall direction. The resultant BNNS/PVA aerogel delivers an ultralow thermal conductivity of 23.5 mW m^−1^ K^−1^ in the transverse direction. The superinsulating aerogel presents both an infrared stealthy capability and a high solar reflectance of 93.8% over the whole sunlight wavelength, far outperforming commercial expanded polystyrene foams with reflective coatings. The anisotropic BNNS/PVA composite aerogel presents great potential for application in energy-saving buildings.

**Supplementary Information:**

The online version contains supplementary material available at 10.1007/s40820-022-00797-6.

## Highlights

- Highly porous aerogel with longitudinally aligned channels and whisker-like ligaments is constructed by solvent-assisted unidirectional freezing.
- The thermal insulation and solar reflection capabilities of the composite aerogel reach a state-of-the-art level.
- The composite aerogel capable of infrared stealth and temperature preservation presents great potential for application in energy-saving buildings.

**Supplementary Information:**

The online version contains supplementary material available at 10.1007/s40820-022-00797-6.

## Graphical abstract

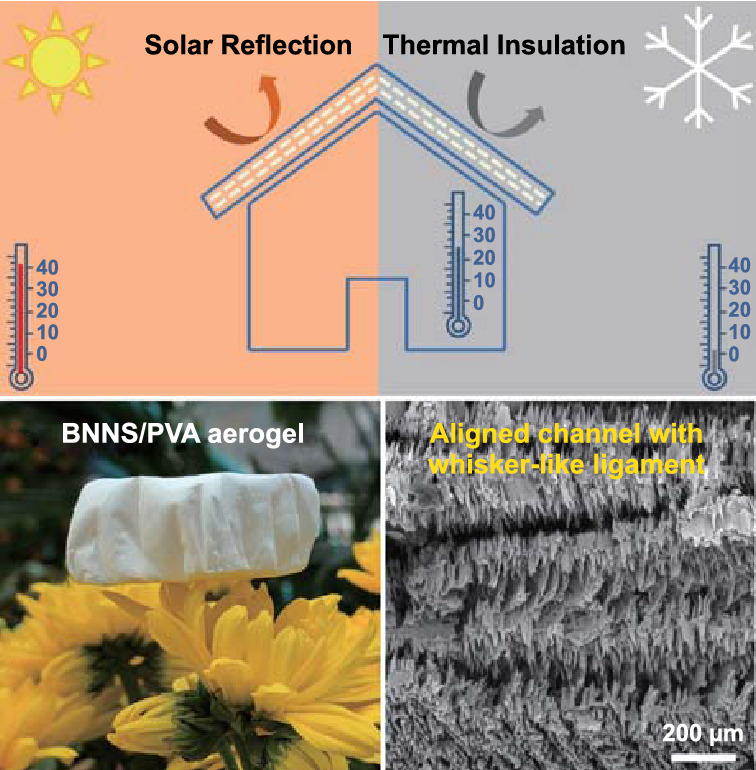

## Introduction

The energy consumption in buildings and houses accounts for a large part of global energy consumption with over 40% in the well-developed regions [1–3]. Vast amounts of energy are required to maintain a comfortable indoor temperature owing to the temperature difference between indoor and outdoor environments. Among all the building components, building envelopes such as roofs and walls are indispensable to construct comfortable, safe, and healthy surroundings for human living. The peak cooling is required in daytime to reduce the higher indoor temperature than the ambient environment stemming from the absorption of sunlight through building envelopes, particularly in hot summer and the tropics with excessive sunshine [4]. Therefore, developing energy-saving building envelopes with super-thermal insulation and high sunlight reflection is of significance for the reduction of building energy consumption, which in turn relieves the overuse of bulky external temperature control systems, such as heating, ventilation, and air conditioning (HVAC), and hence the emission of greenhouse gas [5].

The energy-saving building envelopes currently utilize an approach to integrate thermal insulation materials with reflective coatings that can reduce heat losses to the ambient and mitigate heat gains from the sunlight, respectively. Low-density foams and aerogels with high porosities are commonly developed to achieve thermal insulation. The conventional insulation foams are mainly made from polyurethane (PU) with thermal conductivity (TC) values ranging 20–30 mW m^−1^ K^−1^, aramid (28 mW m^−1^ K^−1^), expanded polystyrene (EPS, 30–40 mW m^−1^ K^−1^), extruded polystyrene (30–40 mW m^−1^ K^−1^), mineral wool (30–40 mW m^−1^ K^−1^), fiberglass (33–44 mW m^−1^ K^−1^), and cork (40–50 mW m^−1^ K^−1^), but these TC values need to be further reduced below 24 mW m^−1^ K^−1^, air’s TC, to achieve super insulation [6–10]. Three-dimensional (3D) aerogels present a great potential to replace these traditional insulation materials because of their high porosities of more than 95% [11]. Silica aerogels exhibit an ultralow TC of ∼ 20 mW m^−1^ K^−1^, but the mechanical brittleness and processing complexity limit their applications in niche fields such as aerospace vehicles [12–14]. In contrast to silica aerogels, polymer aerogels exhibit superior mechanical properties [15], making them promising candidates for practical insulation for building envelopes [16–21].

However, the typically isotropic microstructure of traditional thermal insulation materials is known to be inadequate for highly efficient thermal insulation owing to the ineffectiveness in mitigating the solid heat conduction through the pore walls [22, 23]. To further reduce the solid heat conduction in polymer struts [24, 25], nanosized or nanostructured materials like silica [26], sepiolite nanorods (SEP) [7], graphene oxide (GO) [27], and metal–organic frameworks (MOFs) [28], have been employed as nanofillers to induce phonon scattering at the filler–polymer interfaces such that an even lower TC can be achieved in the composite aerogels. In addition, rational design of anisotropic microstructures, like cellular and lamellar structures, can give rise to further enhanced performance in thermal insulation, in which the heat dissipation can be boosted in the oriented direction, thereby effectively preventing heat localization and reducing heat transfer across the aligned channels in the transverse direction [22, 23]. For example, nanowood with a naturally aligned channel structure presented anisotropic thermal insulation with TCs of 32 mW m^−1^ K^−1^ in the transverse direction and nearly twice (56 mW m^−1^ K^−1^) in the alignment direction [22]. The cellular structure endowed the anisotropic GO/polyimide (PI) nanocomposite with an extremely low transverse TC of 12 mW m^−1^ K^−1^ [23]. Similarly, owing to the well-aligned lamellar structure realized by bidirectional freeze-casting technique, PI/bacterial cellulose hybrid aerogels showed distinct anisotropic thermal insulation performance with TCs of 23 and 44 mW m^−1^ K^−1^ perpendicular to and along the lamella direction, respectively [29].

Despite their excellent thermal insulation, the abovementioned materials seldom shield radiation from the sunlight which radiates heat in the form of electromagnetic waves with wavelengths ranging from 0.3 to 2.5 µm [30]. On the contrary, current thermal insulation materials containing non-white polymers or nanofillers tend to absorb the heat instead of reflecting it [7, 26, 27]. To impart the function of sunlight reflection, a layer of cool-roof paints consisting of white pigments, such as commonly-used TiO_2_ [31], was coated on the surface of the insulating materials [4, 32]. This would require multistep installation procedures and sophisticated constituents, making the retrofitting of existing buildings for energy efficiency rather laborious and costly. Although a few efforts have been devoted to exploring thermal insulation materials with a high solar reflectance, such as nanowood [22] and polyethylene (PE) aerogels [33, 34], their development still remains in its infancy and a formidable challenge, especially for superinsulating aerogels. The challenge mainly stems from the difficulty in achieving desired cell wall structures for simultaneous thermal insulation and broadband solar reflection while maintaining excellent mechanical properties of the aerogels. Although directional freeze-casting has been considered as a promising technique to produce anisotropic aerogels with an ultralow thermal conductivity, the thin cell walls make these aerogels rather fragile while the aligned porous channels with a monotonic pore size cannot afford broadband sunlight reflection. Therefore, it is imperative to develop a new strategy to tune the cell wall structures such that desired multifunctional properties can be achieved in the same aerogel. Most recently, boron nitride (BN) was grown into aerogels [9] with an ultralow TC of 20 mW m^−1^ K^−1^ and was embedded in a polymer matrix to obtain composites [35] with a high solar reflectance of ~ 87%, making it a promising candidate for the production of next-generation superinsulating and solar reflective monoliths.

Herein, an acetone-assisted unidirectional-freezing technique was adopted to produce superinsulating and solar reflective composite aerogels with a cellular structure and tailored cell walls for energy-saving buildings. Water-soluble polyvinyl alcohol (PVA) and BN nanosheets (BNNSs) both in white color were employed to ensure a desirable solar reflectance. Owing to the well-aligned channel structure, the BNNS/PVA composite aerogel exhibited an ultralow TC in the transverse direction. Interestingly, in addition to the construction of aligned microstructures like other solvents [36–38], the presence of acetone generated thick cell walls and reduced the shrinkage, beneficial to the lower density, higher porosity, stronger mechanical performance, and more efficient thermal insulation of the composite aerogels. Furthermore, the whisker-like structure induced by BNNS effectively impeded the heat conduction in the transverse direction without degrading the solar reflectance. The synergistic effect of acetone and BNNS gave rise to an ultralow TC. This work offers a facile approach to fabricate superinsulating aerogels with high solar reflection for energy-saving buildings and other thermal applications.

## Experimental

### Materials and Synthesis of BNNSs

BN powder (99.5%, 325 mesh) and urea were supplied by Alfa Aesar and Aladdin, respectively. PVA and acetone (ACS Reagent) were supplied by Sigma-Aldrich and VWR Chemicals, respectively. Deionized (DI) water was used in the whole process. Although exfoliating bulk BN is more complicated than graphite, a urea-assisted, high-energy ball mill processing has been developed recently to efficiently exfoliate and functionalize BN with a yield of as high as 85% [39, 40]. In view of its high yield and scalability for mass production, the same method was used to prepare BNNSs with hydrophilic amino groups in this work. In brief, BN powder and urea were mixed at a weight ratio of 1:20 in a steel milling container using a planetary ball mill (Changsha Deco Equipment Co., Ltd.) at a rotation speed of 600 rpm for 24 h at room temperature. After ball milling, the mixed powders were dispersed in DI water, followed by centrifugation (Z 326, Hermle Labortechnik GmbH, Germany) at a speed of 5000 rpm for 30 min. The resultant BNNS supernatant was dialyzed for 1 week in DI water to remove urea, and stable few-layer BNNS aqueous dispersion was obtained.

### Fabrication of BNNS/PVA Composite Aerogels

A typical unidirectional freezing method was employed to fabricate anisotropic BNNS/PVA composite aerogels [41, 42]. First, PVA was dissolved in hot water at 90 °C to prepare the polymer solution with a PVA concentration of 2 wt%. Fixed amounts of BNNSs and acetone were dropwise added into the PVA solution, which was poured into a polymer foam mold placed on top of cold source using the apparatus developed previously for unidirectional freeze casting [43]. The temperature of cold source was controlled at –50 °C. The freeze-cast samples were dried on a freeze drier (SCIENTZ-10 N) at a vacuum pressure of less than 5 Pa and a temperature of –56 °C for 1 week to yield BNNS/PVA composite aerogels.

### Characterization

The morphologies of the composite aerogels were characterized using scanning electron microscope (SEM, Hitachi TM3030) at an accelerating voltage of 15 kV. The BNNSs were examined on a Bruker PT scanning probe microscope (Dimension ICON). The Raman spectroscopy (RamanMicro300, Perkin Elmer) was used to confirm the exfoliation of BNNSs. Fourier transform infrared spectroscopy (FT-IR, Bruker Vertex 70 Hyperion 1000) was performed over a wavenumber range of 4000–400 cm^−1^ to examine the difference in chemical compositions between the bulk BN and BNNSs. The mechanical properties were measured in uniaxial compression on a universal testing machine (MTS Alliance RT-5) at a loading rate of 2 mm min^−1^ in accordance with ASTM − C165 − 07. The TCs of aerogels and commercial EPS foams were measured using a hot disk thermal constant analyzer (TPS 2500S, Sweden) based on a transient plane heat source method at room temperature. In view of thermally insulating materials, a large measuring probe with a radius of 6.403 mm was used to minimize the potential heat loss, which was sandwiched between two identical samples. To determine the pore size distributions and porosity of the composite aerogels, the nitrogen gas adsorption/desorption isotherms were obtained on a NOVAtouch, Quantachrome Instruments at 77 K. The degassing was performed at 70 °C, which is lower than the glass transition temperature (*T*_*g*_, 85 °C) and the melting point (*T*_*m*_, 250 °C) of PVA, ensuring that the microstructure of samples was not damaged during degassing. Meanwhile, the vacuum was applied for more than 12 h while degassing. During the analysis, the sample was loaded in the rod-like cell with a diameter of 9 mm. The temperature distribution was recorded using an infrared camera (Fluke Ti25). The reflectance spectra over a wavelength ranging from 0.3 to 2.5 μm were recorded on an ultraviolet–visible-near-infrared (UV–vis-NIR) spectrophotometer (Perkin Elmer Lambda 950). The volumetric shrinkage rates of composite aerogels were calculated based on the sample volumes before and after freeze-drying. The apparent density (*ρ*) was calculated by weighing the aerogels and measuring their volumes. The porosity (*P*) of aerogels was determined by Eq. (1):

1 $$P = \left( {1 - \frac{\rho }{{\rho_{0} }}} \right) \times 100\%$$

where *ρ*_*0*_ is the skeletal density, which is estimated from the weighted average of densities of PVA (1.27 g cm^−3^) and BNNS (2.25 g cm^−3^).

## Results and Discussion

### Structure of BNNS/PVA Composite Aerogels

**Fig. 1:**
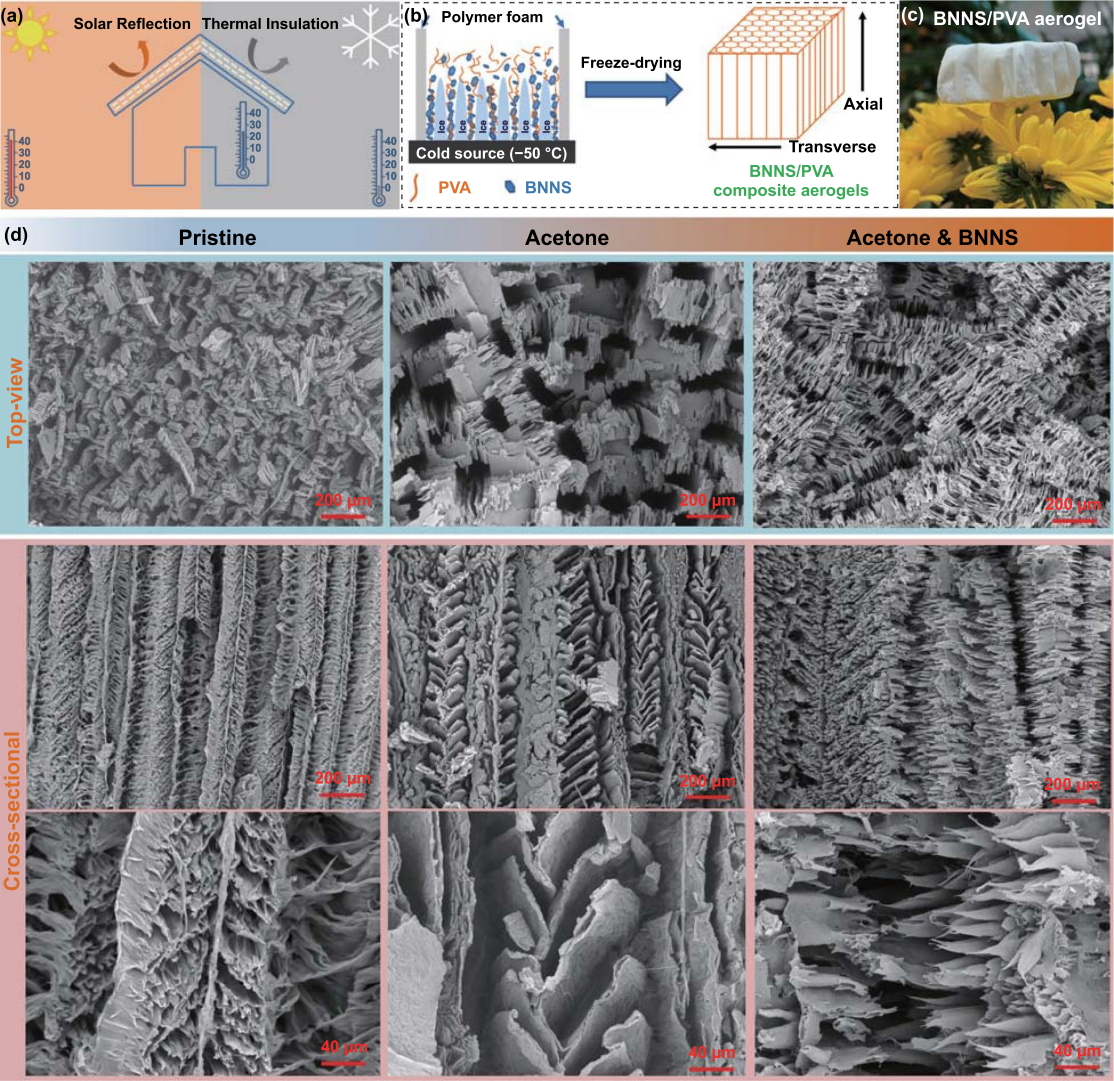
Superinsulating solar-reflective composite aerogels. **a** Schematic illustration showing the temperature regulation of the composite aerogels capable of both thermal insulation and solar reflection for energy-saving buildings. **b** Schematic illustration of the preparation of BNNS/PVA composite aerogels with highly aligned channels through an acetone-assisted unidirectional freezing method. **c** Digital photograph of lightweight BNNS/PVA composite aerogel standing on the petals. **d** SEM images showing microstructure evolution of the BNNS/PVA composite aerogels after adding acetone and BNNS

Lightweight composite aerogels with a well-aligned channel structure were developed to serve as thermal management components in efficient energy-saving buildings, as illustrated in Fig. 1a. The composite aerogel possessed an ultralow TC and a strong solar reflectance that could restrict the heat exchange between inside and outside the building and the solar heat gains when exposed to sunlight, respectively. Therefore, a consistently livable interior temperature range could be maintained for human activities, regardless of high-temperature or low-temperature surroundings. To ensure a high solar reflectance, PVA and BNNS with white appearances are employed as raw materials. The well-aligned channel structure is constructed to boost heat transfer along the oriented direction, while resulting in an ultralow transverse TC.

An acetone-assisted unidirectional freezing method was used to prepare lightweight BNNS/PVA composite aerogels with highly aligned channels, as depicted in Fig. 1b, c. Highly exfoliated BNNSs with hydrophilic amino groups were synthesized by urea-assisted ball-milling (Fig. S1) [39, 40]. The resultant few-layer BNNSs had a lateral dimension of ~ 500 nm and a thickness of ~ 4 nm, as shown by the atomic force microscopy (AFM) image (Fig. S2a-b). The Raman spectra of BNNSs presented a slight upshift of the peak position from 1364 to 1367 cm^−1^ corresponding to the *E*_2g_ mode vibration (Fig. S2c), indicating weakened interlayer interactions in BNNSs and corroborating the exfoliation of bulk BN into few-layer nanosheets [44, 45]. The chemical compositions of exfoliated BNNSs were further investigated using the FT-IR. Along with the two typically characteristic peaks of B-N bonds at 764 and 1335 cm^−1^, two new peaks appeared at 1665 and 3211 cm^−1^ corresponding to the bending and stretching vibrations of -NH_2_ (Fig. S2d), manifesting the successful functionalization of BNNSs with amino groups [39]. These hydrophilic functional groups facilitated stable dispersion of BNNSs in water, as evidenced by the Tyndall effect originating from light scattering (Fig. S2e) [46]. No obvious sediments at the bottom of BNNS dispersions were revealed after standing for two months (Fig. S2f), even for high BNNS concentrations up to 10 mg mL^−1^. The homogenous and stable BNNS dispersions are important for the subsequent fabrication of composite aerogels through a freeze-casting method. As expected, the BNNS/PVA freezing suspensions also exhibited good dispersion in water–acetone mixture (Fig. S3).

The BNNS/PVA composite aerogels were fabricated by dispersing as-prepared BNNSs in water or water–acetone mixture, followed by unidirectional freeze-casting and freeze-drying (Fig. 1b, c). The large temperature difference between the cold source of −50 °C and the room-temperature top of freezing suspension generated a temperature gradient to guide the directional growth of ice crystals. The frozen sample was subsequently freeze-dried to sublimate the ice, yielding a highly porous BNNS/polymer composite aerogel. The top-view and cross-sectional SEM images of the neat PVA aerogel and BNNS/PVA composite aerogels are compared in Fig. 1d. The aerogels exhibited a highly anisotropic cellular structure containing aligned pore channels in the freezing direction, beneficial for generating an ultralow TC transverse to the alignment direction. A close inspection manifests that the addition of acetone and BNNS brought about different morphologies to the composite aerogel. Specifically, the neat PVA aerogel contains aligned cell walls in the freezing direction with abundant ligaments connecting them in the transverse direction (left column) [47]. With the help of acetone which functioned as the antifreeze reducing the freezing rate, the thickness of cell wall increased from ca. 75 to 134 μm on average (mid-column), as shown in Fig. S4 [48, 49]. To demonstrate the general application of acetone, we tried the widely investigated cellulose solution as an additional example. Similarly, after adding 5 vol% acetone in the cellulose solution, the thickness of cell wall of the cellulose aerogel increased, as shown in Fig. S5. Another interesting feature is that after adding BNNS, the aligned cell walls were disconnected to form long hollow channels with dense whisker-like ligaments probably because of the heterogeneous nucleation of ice crystals on BNNS surfaces (right column) [50, 51]. These differences in microstructures are bound to have significant effects on the properties of final composites.

### Physical/Mechanical Properties of BNNS/PVA Composite Aerogels

**Fig. 2:**
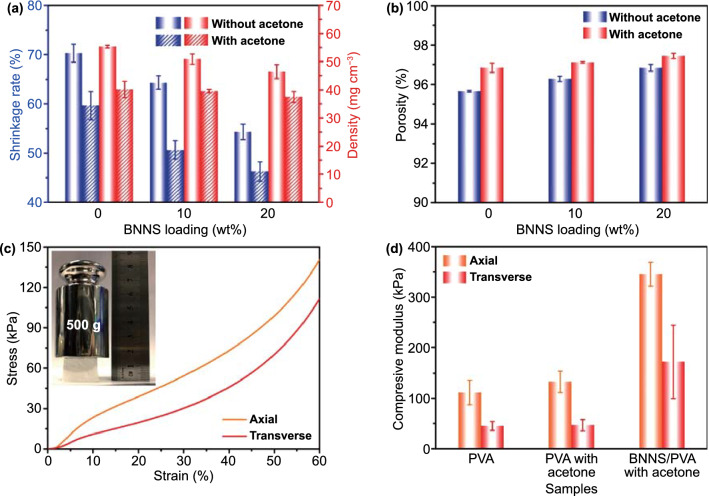
Properties of BNNS/PVA composite aerogels. **a** Shrinkage rate and density and **b** porosity of BNNS/PVA composite aerogels made with or without 5 vol% acetone. **c** Stress–strain curves of BNNS/PVA composite aerogel in the axial and transverse directions (The inset is digital photograph of BNNS/PVA composite aerogel under a dead weight of 500 g). **d** Compressive modulus of aerogels in the axial and transverse directions

Knowing that the TC is closely related to the porosity of the materials and the aerogels fabricated by freeze drying tend to suffer from volume shrinkage [29], the effect of additives on porosity of the resultant composite aerogels was investigated. The shrinkage of the aerogels was obviously mitigated by introducing acetone and BNNS (Fig. S6). With the addition of 5 vol% acetone, the shrinkage rate of PVA aerogel decreased from 70 to 60% (Fig. S7a), a reflection of thickened cell walls due to acetone. After adding 20 wt% BNNSs in the absence of acetone, the shrinkage rate reduced to 54% owing to the supporting effect by BNNSs. Thus, the combination of 5 vol% acetone and 20 wt% BNNS gave rise to the lowest shrinkage rate of 46% for the BNNS/PVA composite aerogel (Fig. 2a). The corresponding density of BNNS/PVA composite aerogels decreased rapidly as the acetone and BNNS loadings increased owing to the reduction in shrinkage. The density of the BNNS/PVA composite aerogel was 32% lower than that of the neat PVA counterpart, *i.e.*, 37.5 vs 55.3 mg cm^−3^, respectively, as shown in Figs. 2a and S7a. It follows then that the porosity increased from 95.6% to 96.8% with the addition of 5 vol% acetone (Fig. S7a), while it continued to increase to 97.4% after introducing 20 wt% BNNSs in the aerogel (Fig. 2b), as a reflection of thickened cell walls and supporting effect by BNNSs.

Lowering the density by adding acetone and BNNS was designed to lower the TC of composite aerogels, but a decrease in density normally translates into deterioration of mechanical performance. Surprisingly, the opposite is actually the case for the BNNS/PVA aerogel where the mechanical properties were significantly improved even at a lower density (Fig. 2c, d). The mechanical properties of the BNNS/PVA aerogel exhibited an anisotropic characteristic with a much higher modulus in the alignment direction than transverse to it (Fig. 2c). The inset in Fig. 2c shows that the BNNS/PVA aerogel was able to withstand over 1900 times its own weight, corroborating its high porosity and compression resistance. Compared to the neat PVA aerogel, the compressive moduli of the BNNS/PVA aerogel were improved by 210% and 280% in the axial and transverse directions, respectively (Fig. 2d), despite a 32% lower density. This was attributed to the thickened cell walls afforded by the acetone and reinforcement of BNNSs. To better indicate the mechanical properties of ultralight materials, the specific compressive modulus defined as the ratio of compressive modulus to apparent density has been calculated, as shown in Fig. S7b. It is clearly seen the synergy arising from the combined acetone and BNNSs is further amplified, delivering remarkable specific compressive moduli over 4.5 times the neat PVA counterparts in both the axial and transverse directions because of concurrently improved modulus and reduced density.

### Thermal Insulation of BNNS/PVA Composite Aerogels

**Fig. 3:**
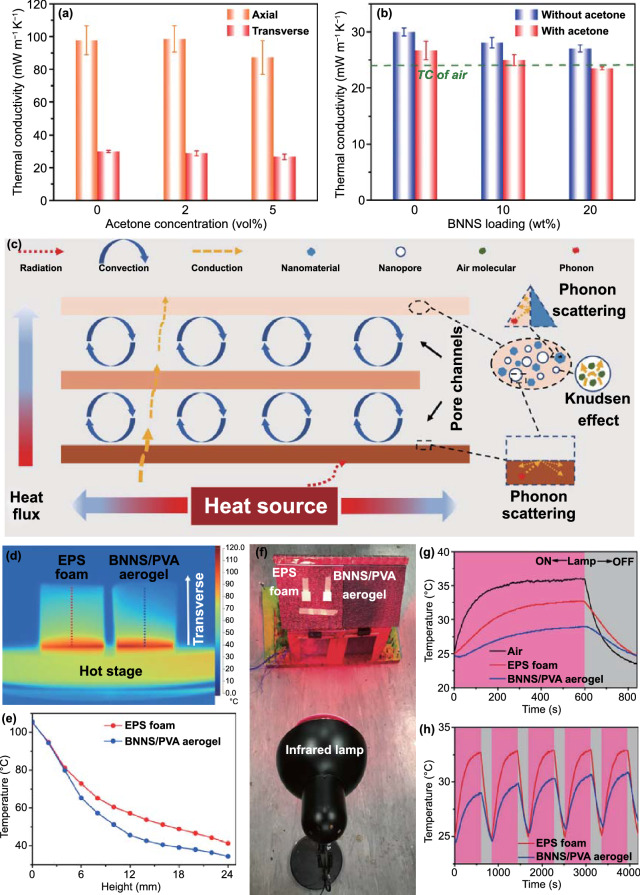
TCs and thermal responses of the aerogels. TCs of **a** the PVA aerogels in the axial and transverse directions prepared with different acetone concentrations and **b** the BNNS/PVA composite aerogels prepared with different loadings of acetone and BNNSs in the transverse direction. **c** Schematic illustration of the heat transfer process and working mechanism of the superinsulating BNNS/PVA composite aerogel. **d** Infrared image and **e** temperature distributions of the BNNS/PVA composite aerogel and EPS foam when placed on a hot stage at 108 °C for 5 min. **f** Setup and **g** temperature changes of the BNNS/PVA composite aerogel and EPS foam when exposed to a non-contact heat source from infrared lamp. **h** Temperature variations of the BNNS/PVA composite aerogel and EPS foam when subjected to five heating and cooling cycles

The anisotropic TCs along and perpendicular to the freezing direction were investigated using the transient plane source (TPS) technique. The aligned structure brought about significant anisotropic thermal-insulating properties of the BNNS/PVA composite aerogels with the TC in the transverse direction being remarkably lower than that in the freezing direction (Figs. 3a, b and S8a). This finding is attributed to the anisotropic cellular networks of the aerogel and partly to possible natural convection along the channel orientation direction, together with the integration of orientation-dependent radiation and solid conduction in the cellular channels [27, 52]. Therefore, the direction transverse to the porous channels is chosen for the thermal insulation application, and the emphasis will be placed on the TCs of aerogels in the transverse direction in the following discussion.

The addition of acetone in the freezing suspensions caused the TC in the transverse direction to decrease from 30.0 to 26.7 mW m^−1^ K^−1^ (Fig. 3a). This reduction is a reflection of the increased porosity originating from a lower shrinkage rate and the reduction of interwall heat conduction [29]. Similarly, a reduction in TC in the alignment direction was also observed after adding 5 vol% acetone (Fig. 3a). The reinforcement of BNNSs saw further reductions in transverse TCs of the BNNS/PVA aerogel regardless of using acetone (Fig. 3b). It is worth noting that with the combined action from both the acetone and BNNSs, the transverse thermal insulation of the aerogel was synergistically enhanced, giving rise to an ultralow TC of 23.5 mW m^−1^ K^−1^ which is even lower than that of air. The synergy arising from the various ameliorating features, such as better aligned structure, higher porosity, disconnected transverse ligaments, as well as the reduction of solid conduction induced by phonon scattering effect between the nanostructured components and polymer matrix [53], was responsible for the remarkable thermal insulation. In contrast, the TCs of the BNNS/PVA composite aerogel in the alignment direction presented an increasing trend with increasing BNNS loading, especially in the presence of acetone (Fig. S8a). The introduction of thermally conductive two-dimensional (2D) BNNSs along the axial direction may explain the observation. The TCs of the state-of-the-art composite aerogels and foams reported in the literature are compared with respect to their densities, as shown in Fig. S8b, presenting the current BNNS/PVA composite aerogel among those having the relatively low TC value. Although a few composite aerogels possessed lower densities and TCs than the current aerogel [7, 23, 27, 54], they involved dark polymers or light absorption materials with rather poor solar reflection.

In an effort to account for the ultralow TC of the composite aerogel, a cartoon showing the synergistic effect of radiation (*λ*_rad_), convection (*λ*_conv_), and conduction ($\lambda_{{{\text{cond}}}}^{g}$ for gas conduction and $\lambda_{{{\text{cond}}}}^{s}$ for solid conduction) on thermal transfer of thermally insulating aerogel is depicted in Fig. 3c [16, 55]. The overall TC of a porous material is calculated by Eq. (2):

2 $$\lambda = \lambda_{{{\text{conv}}}} + \lambda_{{{\text{rad}}}} + \lambda_{{{\text{cond}}}}^{g} + \lambda_{{{\text{cond}}}}^{s}$$

Note that the convection contribution is negligible when the pore size of the insulating materials is below the onset size of natural convection, 1 mm [56, 57], and the radiation contribution is negligible at a relatively low application temperature [26]. In this case, the heat transfer of the thermally insulating materials is determined mainly by the thermal conduction composed of two components, solid and gas conduction [58]. The ultralow TC below the value of air can be correlated to the thermal properties of the nanoscale components and the microstructure in the cell walls [7]. The analysis of nitrogen gas adsorption/desorption isotherms indicates that the cell walls were principally mesoporous with over 20% porosity and an average pore size of 5.4 nm (Fig. S9). The $\lambda_{{{\text{cond}}}}^{g}$ depends on the porosity (*φ*) and pore size (*D*) of the monoliths, which can be expressed by Eqs. (3) and (4) [27, 59, 60]:

3 $$\lambda_{{{\text{cond}}}}^{g} = \frac{{\lambda_{g0} \varphi }}{1 + 2\beta Kn}$$

4 $$Kn = \frac{l}{D}$$

where *λ*_g0_ is the gaseous conductivity in free space (24 mW m^−1^ K^−1^), *Kn* represents the Knudsen number, *l* is the mean free path of a gas molecule (∼70 nm at ambient condition), and the coefficient *β* for air is ~ 2. It can be said that for the BNNS/PVA composite aerogel, a significant reduction in gas conduction occurred in the cell walls because the size of pores in the cell walls was far smaller than the value *l* and the air molecule movement was restricted, known as the Knudsen effect [61]. Meanwhile, the use of nanoscale BNNSs gave rise to a substantial interfacial thermal resistance [25], *i.e.*, Kapitza resistance (*R*_K_) [62], further reducing the solid conduction of the cell walls.

In summary, the highly-aligned porous channels, high porosity, mesoporous cell walls, and high interfacial thermal resistance between BNNS and PVA synergically contributed to the ultralow TC of 23.5 mW m^−1^ K^−1^ of the aerogel in the transverse direction. First, the anisotropic structure gave rise to boosted heat dissipation in the alignment direction [22], thereby effectively preventing heat localization and reducing the heat transfer across the aligned channels in the transverse direction. Second, the gas conduction dominated the TC of aerogel owing to the high porosity of 97.4% [63]. Third, the mesopores with far smaller sizes than the mean free path of air significantly reduced the gas conduction in the cell walls [64]. Last, BNNSs were employed as nanofillers to induce phonon scattering at the BNNS-PVA interfaces [25], further reducing the solid heat conduction in the composite cell walls.

The anisotropic heat insulation property of composite aerogels in different directions was vividly visualized by thermal infrared images. Two samples with vertical and horizontal channels were put on a hot stage at 108 °C for 12 min, followed by recording the temperature distribution along the sample height, revealing a large difference in the two directions (Fig. S10). The temperature along the transverse direction was generally lower than that in the orientation direction at the same height, confirming better thermal insulation performance in the transverse direction. A similar comparison was made of the temperature distributions between the BNNS/PVA composite aerogel and commercial EPS foam with a TC of 30.5 mW m^−1^ K^−1^ following the similar procedure. Worthy of note is that the samples were placed in the center to reduce the influence of uneven temperature distribution of the hot stage with the temperature at the edge being much lower than at the center, as shown in the top-view infrared image in Fig. S11. The side-view infrared image and the corresponding temperature distributions at different heights are shown in Fig. 3d, e. The BNNS/PVA aerogel delivered a generally lower temperature distribution outperforming the EPS foam in thermal insulation. In addition to the use of hot stage as contact heat source, an infrared lamp (IR 250 RH IR2 250 W, Philips) was applied as non-contact heat source (Fig. 3f). Both the EPS foam and the BNNS/PVA aerogel were attached on the roof of a model house and the temperatures at the back of the two materials were monitored. Once the light was ‘on’, the temperature increase behind the EPS foam was far more significant and faster than that behind the BNNS/PVA aerogel. After heating for 10 min, the temperature at the back of EPS foam was ~ 3 °C higher than that of the composite aerogel while the cooling was also faster in the former (Fig. 3g). Besides, the composite aerogel exhibited good stability when experiencing five heating and cooling cycles (Fig. 3h). Regardless of contact and non-contact heat sources, the composite aerogel delivered superior thermal insulation performance, presenting promising applications in thermal protection [65].

**Fig. 4:**
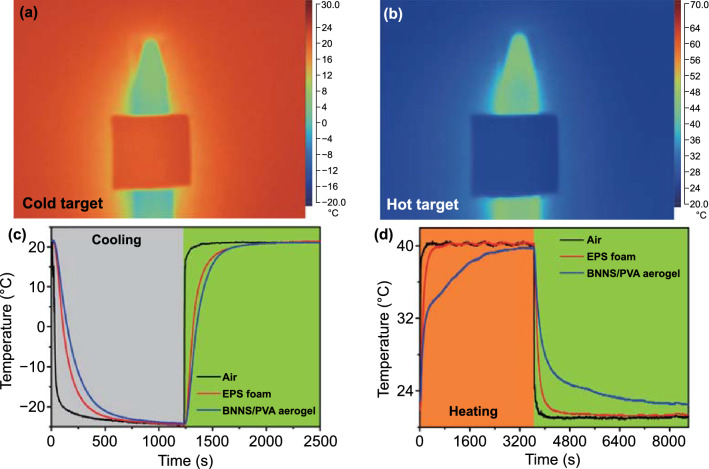
Superinsulating BNNS/PVA composite aerogels for infrared stealth and temperature preservation. Infrared images showing stealthy performance of the BNNS/PVA composite aerogel on **a** cold and **b** hot targets. Temperature evolution of the BNNS/PVA composite aerogel, EPS foam and air when exposed to **c** cold (fridge) and **d** hot (oven) environments

The superior thermal insulation can endow the products with an infrared stealthy function, preventing the targets with a relatively low or high temperature from detection by a sensitive infrared apparatus [66–69]. Figure 4a, b demonstrates that when a cold or hot target was covered by a piece of 9-mm-thick BNNS/PVA composite aerogel, the color of the covered area was consistent with the ambient color, while the remaining area of the uncovered target maintained a low or high temperature. The infrared stealthy function of the composite aerogel makes the target difficult to be distinguished under an infrared detection device owing to excellent thermal insulation. The better thermal insulation of the composite aerogel than the EPS foam corroborates with their infrared stealthy performance (Fig. S12a-b), which could be further improved by applying multilayer materials or increasing the thickness (Fig. S12c-d). The superinsulating performance and infrared stealthy function of the BNNS/PVA composite aerogel may find potential applications in next-generation military equipment.

In addition to the infrared stealthy function, the aerogel with thermal superinsulation also exhibited a unique capability to preserve temperature for drug transport, food storage, and extreme environment scenarios such as polar region and outer space [18]. A parallel experiment was performed to demonstrate the above potential using commercial EPS foam as a control. Cubic boxes with 48 mm in length, 28 mm in width, and 30 mm in height were assembled using several pieces of ~ 9 mm thick EPS foams and composite aerogels. Then, the boxes made of different insulating materials were placed in cold (at –25 °C in a fridge) and hot (at 40 °C in an oven) environments to record real-time inner temperature changes using thermocouples. The aerogel box exhibited more gradual temperature changes than the EPS foam counterpart in both cold (Fig. 4c) and hot (Fig. 4d) environments, confirming its excellent capability to preserve the temperature inside the box.

### Solar Reflectance of BNNS/PVA Composite Aerogels

**Fig. 5:**
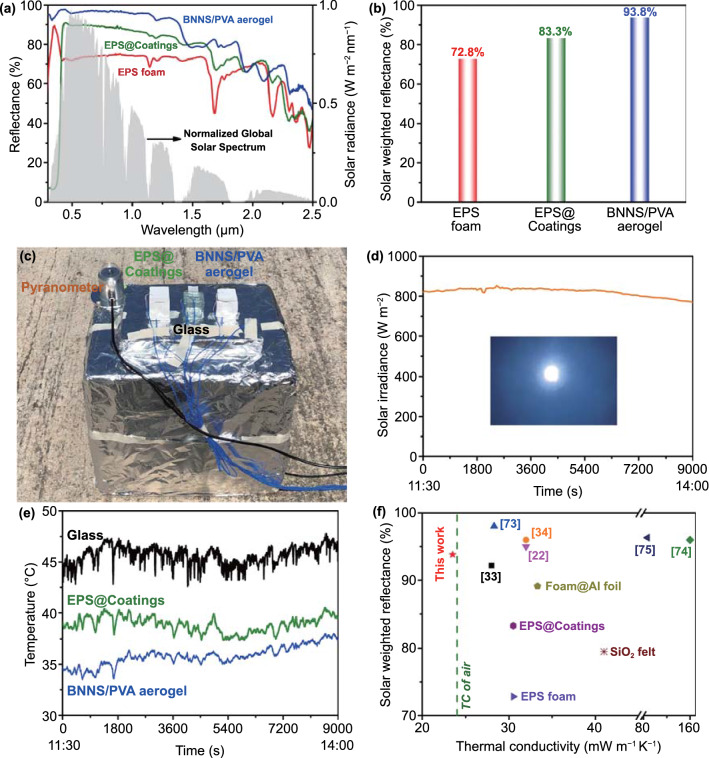
Superinsulating solar-reflection BNNS/PVA composite aerogel for energy-saving buildings. **a** UV–vis-NIR spectra of 9-mm-thick BNNS/PVA composite aerogel, EPS foam and EPS@Coatings presented against normalized ASTM G173 global solar spectrum. **b** Solar weighted reflectance of the BNNS/PVA composite aerogel, EPS foam and EPS@Coatings. **c** Setup, **d** real-time solar irradiance with the real-time weather condition in inset, and **e** real-time temperature curves inside the boxes assembled using the BNNS/PVA composite aerogel, EPS@Coatings and glass when exposed to sunlight (Mostly sunny, July 09, 2021 in Hong Kong). **f** Comparison of solar reflectance performance with respect to TC of the BNNS/PVA composite aerogel with thermally insulating materials reported in the literature, including PE aerogels [33, 34, 73], nanowood [22], cooling wood [74], and PVA porous film [75], and commercial products including neat EPS foam, EPS@Coatings, SiO_2_ felt, and foam@Al foil

When using the thermal insulation aerogels for energy-saving buildings, another important parameter, namely the solar reflectance, needs to be taken into account. The white appearance of the porous composite aerogels with high porosities ensured relatively high solar reflectance. The 9-mm-thick BNNS/PVA aerogel presented a generally higher reflectance over the whole solar wavelengths from 0.3 to 2.5 μm than the EPS foam and EPS@Coatings counterparts according to the UV–vis-NIR spectra shown in Fig. 5a. The EPS@Coatings is another commercial product prepared by applying reflective coatings on the neat EPS foam. The hierarchical micro- and nanostructures with mesopores of sizes broadly distributed 3–20 nm (Figs. 1d and S9) were responsible for the excellent sunlight scattering by the composite aerogel [30, 70]. Considering that the solar intensity varies with the wavelength, the solar intensity weighted reflectance, $\overline{R}$, was calculated by Eq. (5) [30, 71]:

5 $$\overline{R} = \frac{{\mathop \smallint \nolimits_{{\lambda_{1} }}^{{\lambda_{2} }} I_{{{\text{solar}}}} \left( \lambda \right)R\left( \lambda \right){\text{d}}\lambda }}{{\mathop \smallint \nolimits_{{\lambda_{1} }}^{{\lambda_{2} }} I_{{{\text{solar}}}} \left( \lambda \right){\text{d}}\lambda }}$$

which is defined as the ratio of the reflected solar intensity within a certain wavelength range (from *λ*_1_ to *λ*_2_) to the total incident solar intensity in the same range according to the UV–vis-NIR spectral reflectance, *R*(*λ*), of the BNNS/PVA aerogel and normalized ASTM G173 global solar intensity spectrum, *I*_solar_(*λ*). The whole solar wavelengths from 0.3 to 2.5 μm were considered in this work, defining λ_1_ = 0.3 and λ_2_ = 2.5 in the formula. The integration of the product of *I*_*solar*_(*λ*) and *R*(*λ*) was calculated, followed by calculating the integration of *I*_*solar*_(*λ*), giving rise to the ratio of these two sums as the solar weighted reflectance. It is remarkable that the weighted reflectance of the composite aerogel reached up to 93.8% over the whole wavelength range, which is much higher than those of the neat EPS foam and EPS@Costings, as shown in Fig. 5b.

With practical applications of the composite aerogels in mind, a field test was performed on a hot summer day (mostly sunny on July 09, 2021) in Hong Kong to confirm the cooling effect of the composite aerogel when used as building materials. The details of setup are shown in Fig. 5c. Three cubic boxes with 48 mm in length, 28 mm in width, and 30 mm in height were assembled of glass, the EPS@Coatings and the composite aerogel, which were mounted on a 0.3 m thick large foam platform covered with aluminum (Al) foil for the isolation of thermal conduction from the ground and the reduction of solar absorption of the testing boxes [72]. Thermocouples were used to monitor in real-time the internal temperature of the boxes under sunlight irradiation (Fig. 5d). The temperature evolution curves measured in the field test (Fig. 5e) indicate that the temperatures in the boxes were higher in the descending order of glass, EPS@Coatings, and composite aerogel. It is interesting to note that even after two and a half hours of exposure to sunlight (from 11:30 am to 14:00 pm under sunlight irradiation of ~ 800 W m^−2^) on a mostly sunny day, the temperature distribution in the composite aerogel remained lower than those in the glass and EPS@Coatings. The average temperature of the BNNS/PVA composite aerogel was ~ 10 and 3 °C lower than the glass and EPS@Coatings counterparts, which are equivalent to 22% and 8% reductions, respectively. A lower temperature inside the composite aerogel box under the sunlight signifies less external input energy required to cool down the interior of a building on a hot day [73]. Overall, the BNNS/PVA composite aerogel developed in this work delivered a combination of excellent thermal insulation and high solar reflection performance, offering significant energy savings as a dual-functional building envelope. In comparison to other commercial products and insulating materials reported in the literature, both the thermal insulation and solar reflection capabilities of the composite aerogel reached a state-of-the-art level, as shown in Fig. 5f. For large-scale applications in buildings, large-size aerogels with horizontal pore alignments are required. A proof-of-concept design for a novel block-by-block freezing set-up was proposed (see Supplementary Information for details), demonstrating the possibility of producing large-size BNNS/PVA aerogels for practical applications (Fig. S13). Further developments of optimized mass production process of BNNSs, freezing set-up and energy-efficient drying technology are essential to delivering a truly energy-efficient building envelope from production to application.

## Conclusions

In summary, a highly anisotropic and lightweight BNNS/PVA composite aerogel with excellent thermal insulation and high solar reflection was produced through a solvent-assisted unidirectional freezing method. The coupling of thickened cell wall induced by acetone and the reinforcement from nanoscale BNNSs effectively reduced the shrinkage rate of aerogels from 70 to 46%, giving rise to an ultralow density of 37.5 mg cm^−3^ and a high porosity of 97.4%. The presence of nanostructured BNNSs disconnected the transverse ligaments between the adjacent parallel cell walls, further reducing the heat conduction in the transverse direction and bringing about anisotropic thermal conduction. Thus, the final BNNS/PVA composite aerogel possessed an ultralow TC of 23.5 mW m^−1^ K^−1^, which is even lower than that of the air. The excellent thermal insulation endowed the aerogel with capabilities of simultaneous infrared stealth and temperature preservation. The superinsulating aerogel maintained a high solar weighted reflectance of 93.8% over the whole sunlight wavelength. The energy-saving effect of aerogel was demonstrated by real-time temperature measurements, showing much less heat gains than commercial EPS foams with reflective coatings and maintaining a temperature ~ 10 °C lower than the glass in a field test on a hot sunny day. The superinsulating composite aerogel equipped with a high solar reflection capability enables a substantial reduction in energy consumption for cooling when exposed to sunlight, offering great potential for use in future ecofriendly and energy-saving buildings.

## Supplementary Information

Below is the link to the electronic supplementary material.

Supplementary file1 (PDF 1091 KB)
