# Supplementary material for: Superinsulating BNNS/PVA Composite Aerogels with High Solar Reflectance for Energy-Efficient Buildings
Source: Nanomicro Lett. 2022 Feb 2;14:54. doi: 10.1007/s40820-022-00797-6 (PMC8811070; doi:10.1007/s40820-022-00797-6)
Supplement: Supplementary file 1 — Supplementary file1 (PDF 1091 KB) [file 40820_2022_797_MOESM1_ESM.pdf]

Supporting Information for

Superinsulating BNNS/PVA Composite Aerogels with High Solar Reflectance for Energy-Efficient Buildings

Jie Yang[1], Kit-Ying Chan[1], Harun Venkatesan[1], Eunyoung Kim[1], Miracle Hope Adegun[1], Jeng-Hun Lee[1], Xi Shen[1, 2, *], Jang-Kyo Kim[1, *]

[1]Department of Mechanical and Aerospace Engineering, The Hong Kong University of Science and Technology, Clear Water Bay, Kowloon, Hong Kong, P. R. China

[2]Department of Aeronautical and Aviation Engineering, The Hong Kong Polytechnic University, Hung Hom, Kowloon, Hong Kong, P. R. China

*Corresponding authors. E-mail: xi.shen@polyu.edu.hk (Xi Shen); mejkkim@ust.hk (Jang-Kyo Kim)

S1 Preparation of Large-scale BNNS/PVA Composite Aerogel

The preparation of large-size products having horizontal pore alignment is challenging based on the ice-templating method due to the conventional bottom-up freezing set-up (**Fig. 1**b). To tackle the technological challenge, the position of the cold source was switched from the bottom to the side so as to prepare the aligned aerogels with horizontal channels (**Fig. S13**a). Compared to the bottom cold source, the drying time can be shortened when using the side cold source to fabricate the thin sample required for applications. It is worth mentioning that the pore alignment in the aerogel fabricated by the unidirectional freezing method with the side cold source could only be maintained below a limited distance, beyond which the pores tend to become randomly-oriented because of the increasing solidification front temperature. To maintain a consistent pore alignment across the whole structure, a novel block-by-block freeze-casting technique was devised by moving the cold source (**Fig. S13**b). In this technique, a movable stainless-steel reservoir filled with liquid nitrogen was used as the cold source and put in a Teflon mold. The initial distance between the cold source and the other side of the Teflon mold was set at around 1.5 cm. The precursor dispersion was poured into the mold so that it became frozen driven by the temperature gradient between the cold reservoir and the side of the Teflon mold. Once freezing was completed, the cold source was moved in the direction opposite to freezing by 1.5 cm. A new batch of dispersion was poured into the mold and freeze-cast to form a new block interconnected with the initial frozen one. After repeating the above freeze-casting procedure and freeze-drying, a large-size BNNS/PVA aerogel with a lateral dimension of 20×20 cm^2 and a thickness of about 8.5 mm was obtained, as shown in **Fig. S13**c. Further optimization of this technique in terms of continuous production, seamless bonds between the adjacent blocks, and enhanced and more economical drying process, *e.g.*, using an automatic moving cold source and ambient drying technique, is necessary to achieve desired optical and thermal properties of large-size aerogels.

S2 Supplementary Results

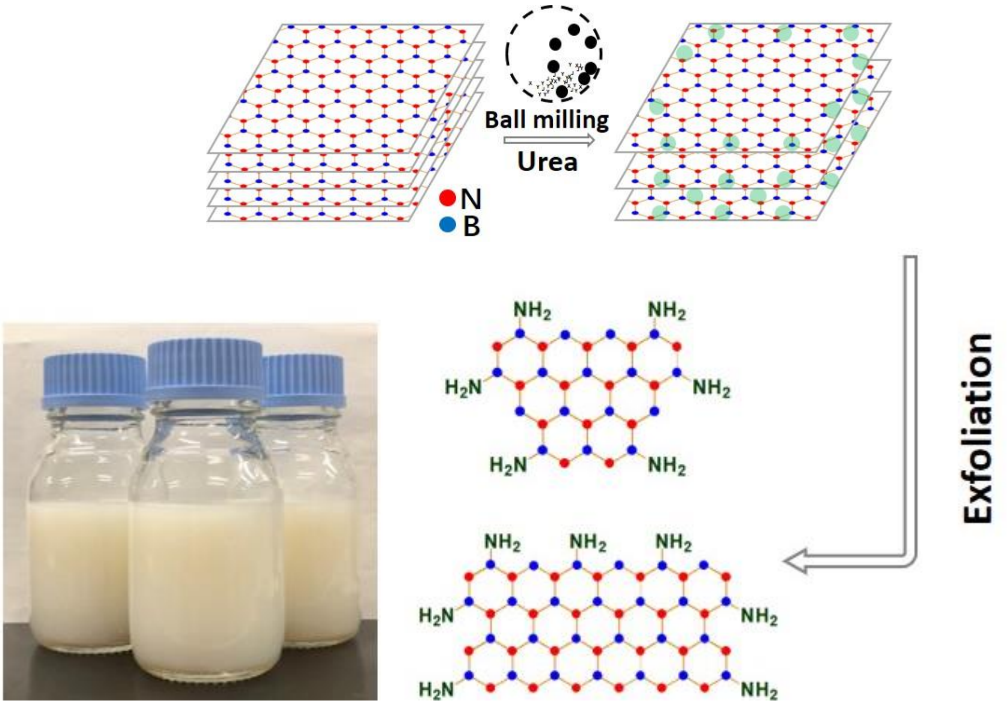

Fig. S1 Schematic of the urea-assisted ball-milling method to fabricate few-layer BNNSs with an optical image showing a high yield

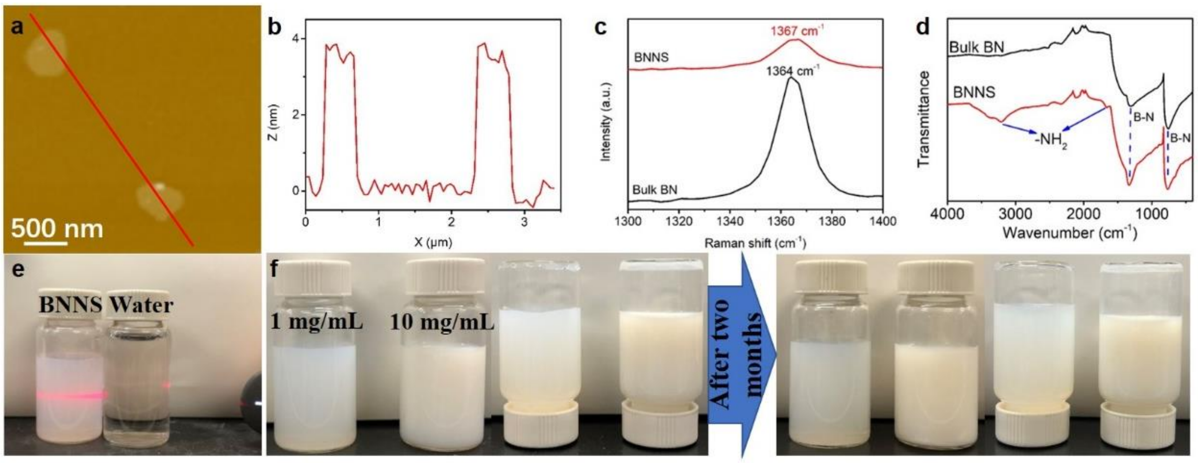

Fig. S2 Morphology, chemical composition and dispersion of BNNSs. **a** AFM image with **b** corresponding height profile of BNNSs. **c** Raman and **d** FITR spectra of bulk BN and BNNSs. Optical images showing **e** the Tyndall effect and **f** excellent aqueous dispersions with different BNNS concentrations

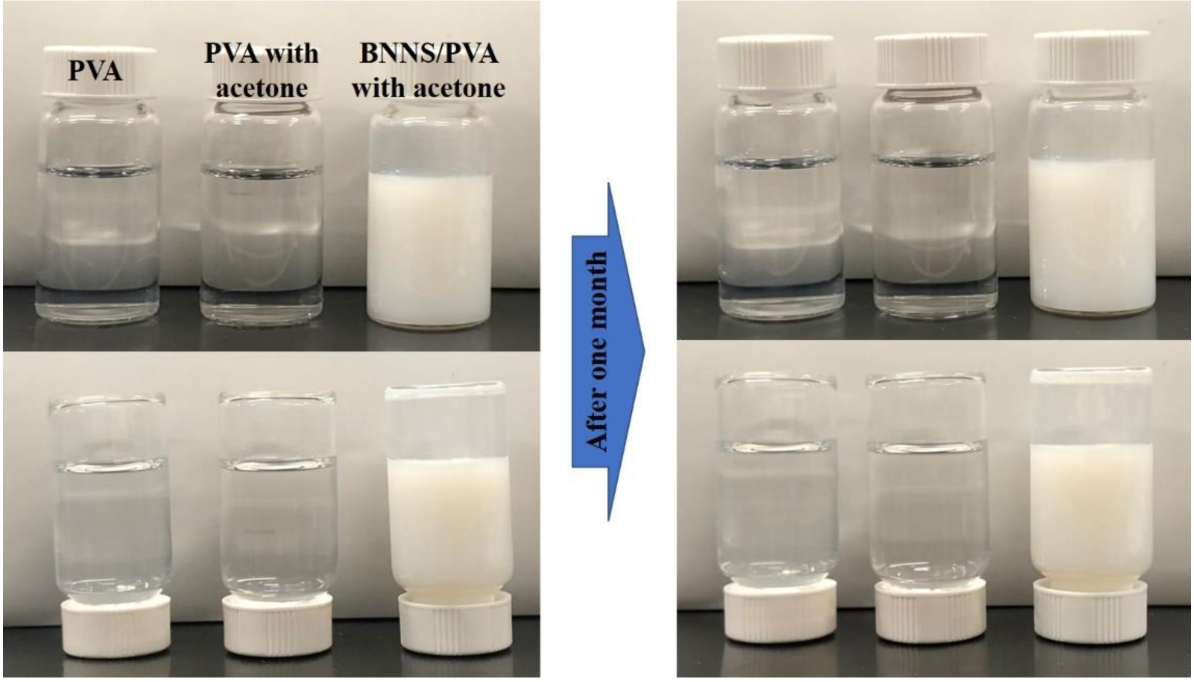

Fig. S3 Optical images showing the dispersion of BNNSs in PVA solution with acetone

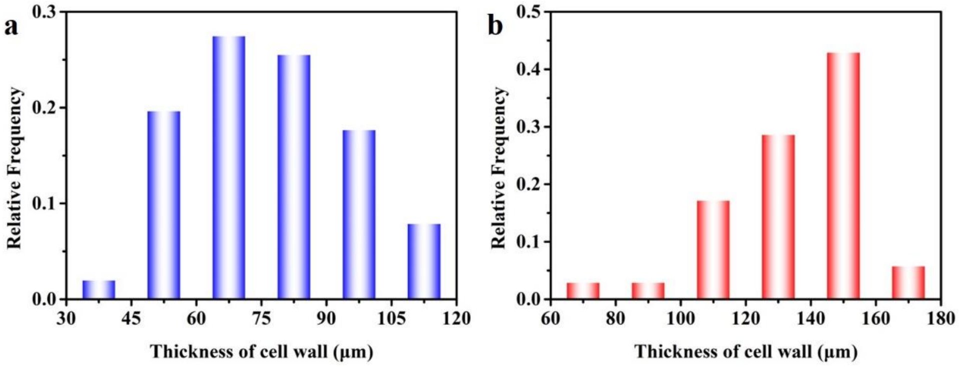

Fig. S4 Thickness distribution of PVA aerogels made **a** without and **b** with acetone

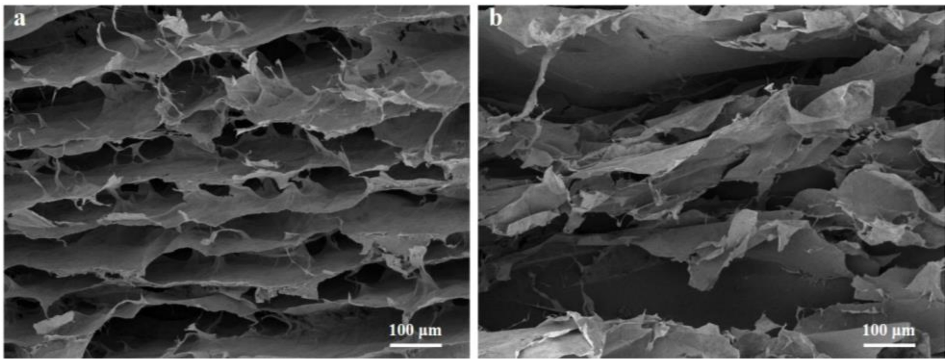

Fig. S5 SEM images of cellulose aerogels made **a** without and **b** with acetone

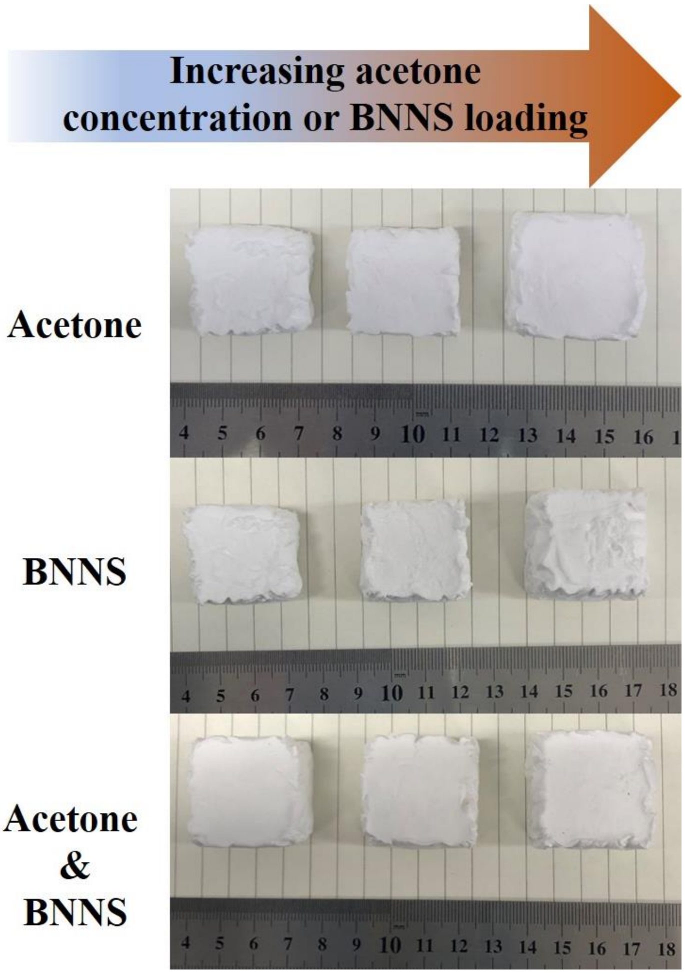

Fig. S6 Optical images showing the sizes of the resultant aerogels prepared with PVA solutions containing different acetone concentrations and/or BNNS loadings

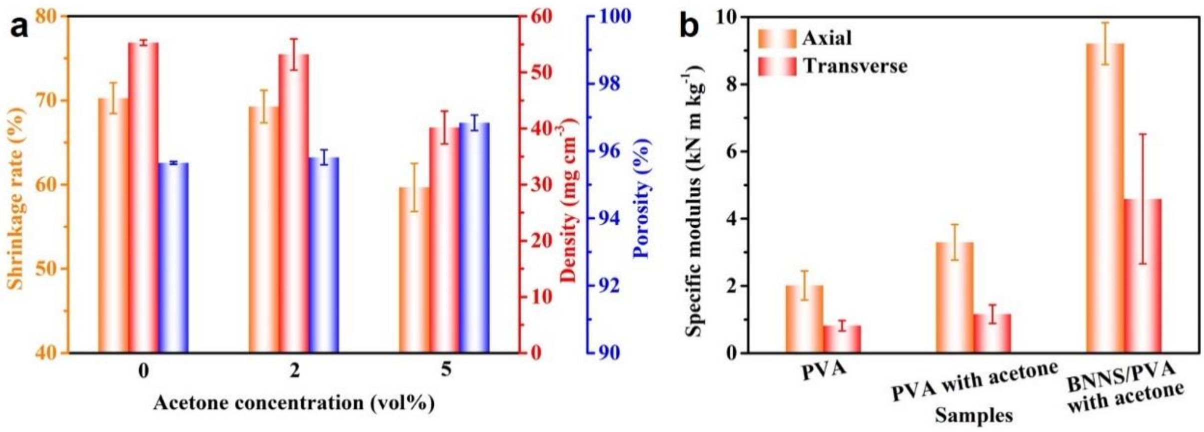

Fig. S7 a Shrinkage rate, density, and porosity of PVA aerogels with increasing acetone concentration. **b** Specific compressive moduli of aerogels in the axial and transverse directions

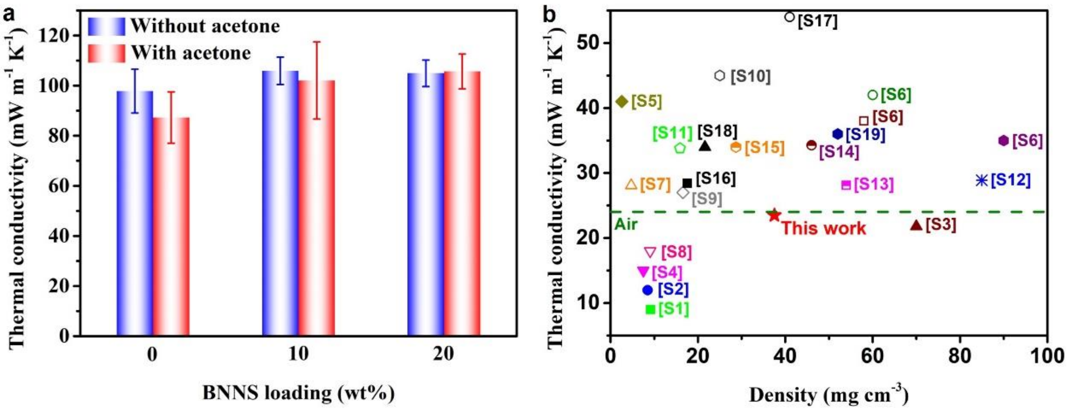

Fig. S8 a Axial thermal conductivity of the BNNS/PVA composite aerogels made with or without acetone. **b** Comparison of thermal conductivities with respect to densities of the current BNNS/PVA composite aerogel in the transverse direction and state-of-the-art composite aerogels or foams reported in the literature: anisotropic reduced graphene oxide (rGO)/polyimide (PI) aerogels [S1, S2], isotropic SiO_2/PI aerogel [S3], anisotropic GO/sepiolite nanorod (SEP)/cellulose nanofiber (CNF) aerogel [S4], anisotropic metal–organic framework (MOF)/CNF aerogel [S5], anisotropic SiO_2/Silk fibroin aerogel [S6], anisotropic SEP/Silk fibroin aerogel [S6], anisotropic MXene/Silk fibroin aerogel [S6], isotropic MoS_2/CNF aerogel [S7], anisotropic zirconium phosphate (ZrP)/rGO/CNF aerogel [S8], anisotropic organosilica/CNF aerogel [S9], isotropic alolt/sodium silicate/CNF aerogel [S10], isotropic sepiolite clay/CNF aerogel [S11], anisotropic GO/phenol-formaldehyde wood [S12], anisotropic hydroxyapatite/chitosan aerogel [S13], isotropic attapulgite/gelatin aerogel [S14], anisotropic clay/cationic amylopectin aerogel [S15], anisotropic BNNS/CNF aerogel [S16], isotropic montmorillonite/cellulose aerogel [S17], isotropic silver nanowires (AgNWs)/Fe_3O_4/melamine-formaldehyde (MF) foam [S18], and isotropic layered double hydroxide (LDH)/GO/PI aerogel [S19]

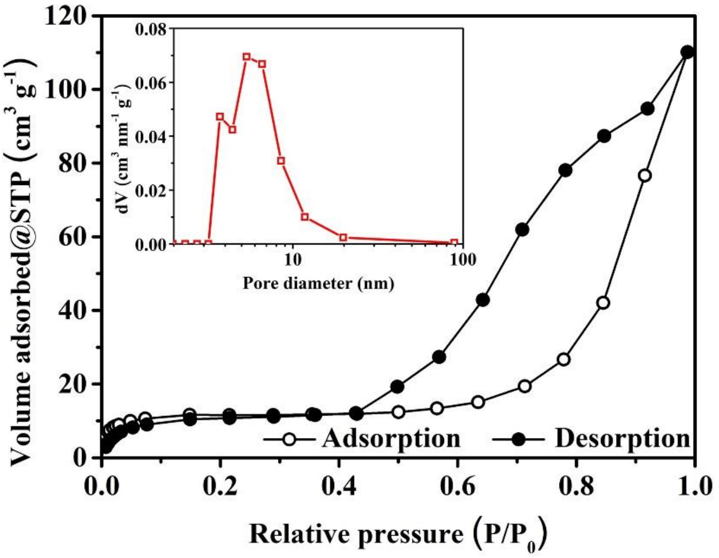

Fig. S9 Brunner−Emmet−Teller (BET) analysis based on a Quantachrome Instruments (NOVAtouch, USA) at 77 K, and nitrogen adsorption (unfilled circles) and desorption (filled circles) isotherms of the BNNS/PVA composite aerogel. The inset shows the pore size distribution larger than 2 nm obtained using the Barrett−Joyner−Halenda (BJH) method according to the desorption branch of the isotherm. The BJH analysis reveals mesopores with diameters ranging 3−20 nm while the total pore volume is about 0.21 $cm^3\ g^{-1}$

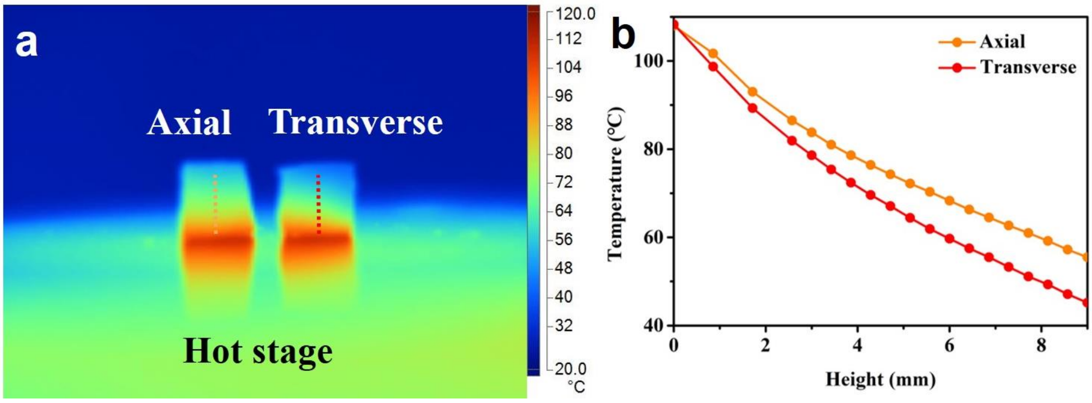

Fig. S10 **a** Infrared image and **b** corresponding temperature profiles of the BNNS/PVA composite aerogel in the axial and transverse directions when placed on a hot stage at 108 °C for 12 min

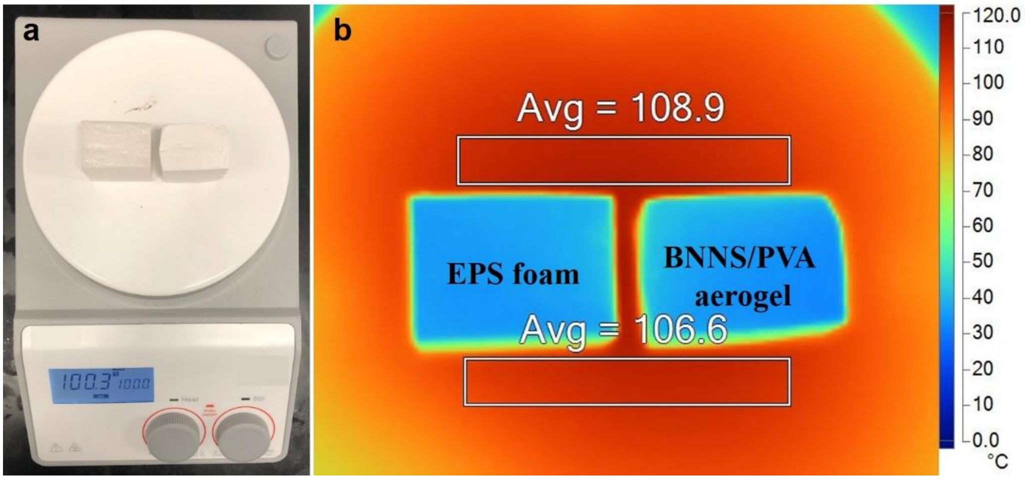

Fig. S11 **a** Photograph and **b** corresponding top-view infrared image of the BNNS/PVA composite aerogel and EPS foam when placed on a hot stage

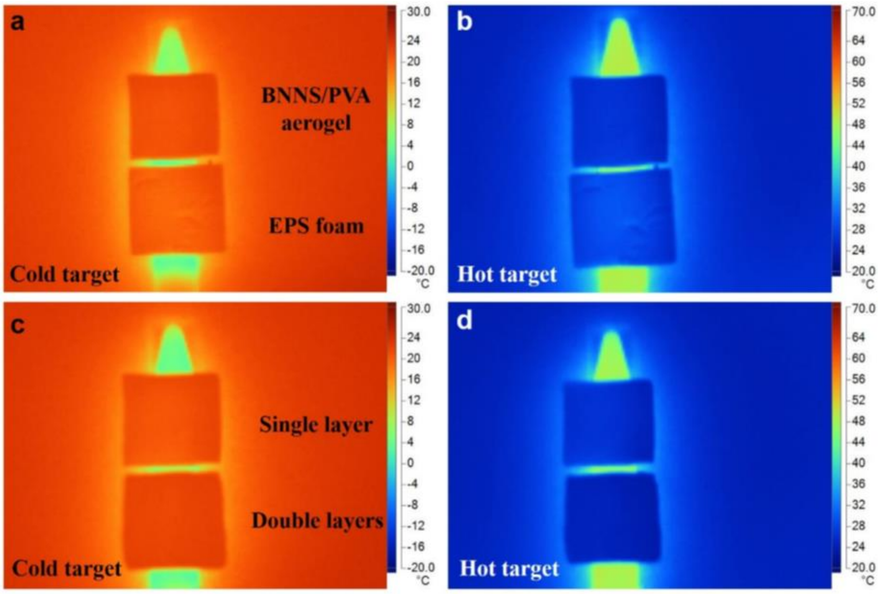

Fig. S12 Infrared images showing the infrared stealthy performance of **a-b** the BNNS/PVA composite aerogel on cold and hot targets compared to commercial EPS foam and **c-d** the double-layered BNNS/PVA composite aerogel

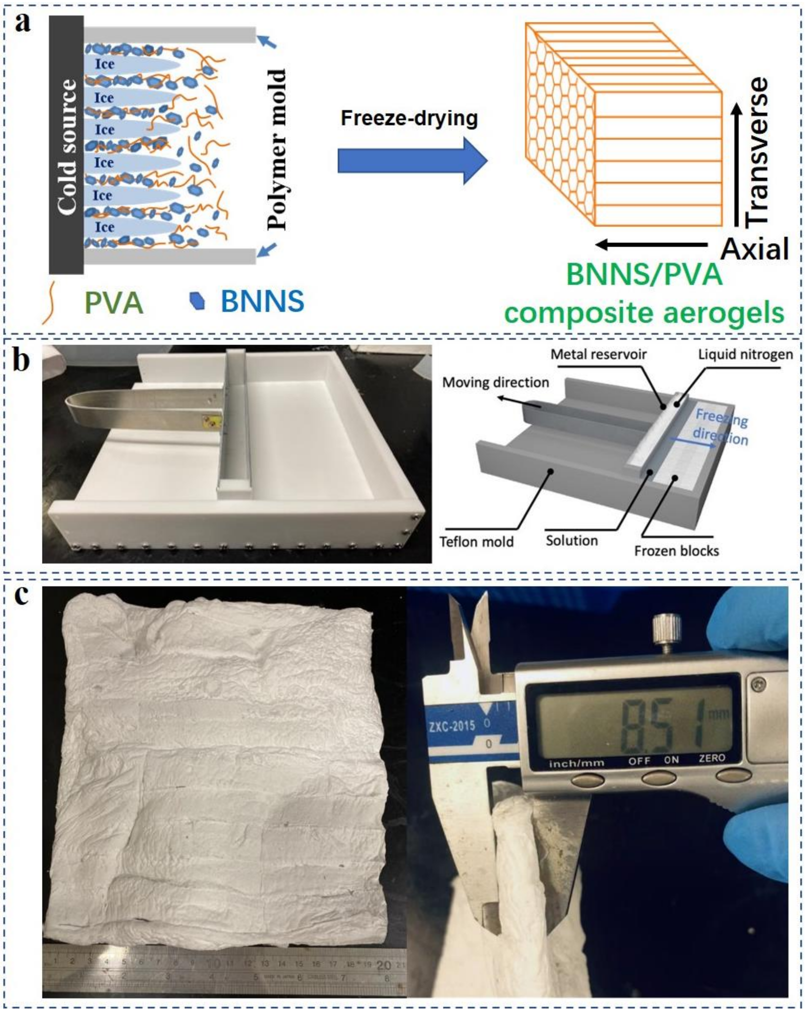

Fig. S13 a Schematic illustration of the preparation of aligned aerogels with horizontal channels through a unidirectional freezing method with the side cold source, **b** set-up of a novel block-by-block freeze-casting technique, and **c** photographs of large-size BNNS/PVA composite aerogel with a thickness of about 8.5 mm

Supplementary References

[S1] Q. Peng, Y. Qin, X. Zhao, X. Sun, Q. Chen et al., Superlight, mechanically flexible, thermally superinsulating, and antifrosting anisotropic nanocomposite foam based on hierarchical graphene oxide assembly. ACS Appl. Mater. Interfaces **9**(50), 44010-44017 (2017). https://doi.org/10.1021/acsami.7b14604

[S2] Y. Qin, Q. Peng, Y. Zhu, X. Zhao, Z. Lin et al., Lightweight, mechanically flexible and thermally superinsulating rGO/polyimide nanocomposite foam with an anisotropic microstructure. Nanoscale Adv. **1**(12), 4895-4903 (2019). https://doi.org/10.1039/c9na00444k

[S3] W. Fan, X. Zhang, Y. Zhang, Y. Zhang, T. Liu, Lightweight, strong, and super-thermal insulating polyimide composite aerogels under high temperature. Compos. Sci. Technol. **173**, 47-52 (2019). https://doi.org/10.1016/j.compscitech.2019.01.025

[S4] B. Wicklein, A. Kocjan, G. Salazar-Alvarez, F. Carosio, G. Camino et al., Thermally insulating and fire-retardant lightweight anisotropic foams based on nanocellulose and graphene oxide. Nat. Nanotechnol. **10**(3), 277-283 (2014). https://doi.org/10.1038/nnano.2014.248

[S5] S. Zhou, V. Apostolopoulou-Kalkavoura, M.V.T. Costa, L. Bergström, M. Strømme et al., Elastic aerogels of cellulose nanofibers@metal–organic frameworks for thermal insulation and fire retardancy. Nano-Micro Lett. **12**, 9 (2020). https://doi.org/10.1007/s40820-019-0343-4

[S6] H. Maleki, T. Fischer, C. Bohr, J. Auer, S. Mathur et al., Hierarchically organized biomimetic architectured silk fibroin-ceramic-based anisotropic hybrid aerogels for thermal energy management. Biomacromolecules **22**(4), 1739-1751 (2021). https://doi.org/10.1021/acs.biomac.1c00175

[S7] L. Yang, A. Mukhopadhyay, Y. Jiao, Q. Yong, L. Chen et al., Ultralight, highly thermally insulating and fire resistant aerogel by encapsulating cellulose nanofibers with two-dimensional MoS_2. Nanoscale **9**(32), 11452-11462 (2017). https://doi.org/10.1039/c7nr02243c

[S8] D. Wang, H. Peng, B. Yu, K. Zhou, H. Pan et al., Biomimetic structural cellulose nanofiber aerogels with exceptional mechanical, flame-retardant and thermal-insulating properties. Chem. Eng. J. **389**, 124449 (2020). https://doi.org/10.1016/j.cej.2020.124449

[S9] D. Wang, X. Feng, L. Zhang, M. Li, M. Liu et al., Cyclotriphosphazene-bridged periodic mesoporous organosilica-integrated cellulose nanofiber anisotropic foam with highly flame-retardant and thermally insulating properties. Chem. Eng. J. **375**, 121933 (2019). https://doi.org/10.1016/j.cej.2019.121933

[S10] S. Gorgieva, U. Jančič, S. Hribernik, D. Fakin, K.S. Kleinschek et al., Processing and functional assessment of anisotropic cellulose nanofibril/alolt/sodium silicate: based aerogels as flame retardant thermal insulators. Cellulose **27**(3), 1661-1683 (2019). https://doi.org/10.1007/s10570-019-02901-3

[S11] P. Gupta, C. Verma, P.K. Maji, Flame retardant and thermally insulating clay based aerogel facilitated by cellulose nanofibers. J. Supercrit. Fluids **152**, 104537 (2019). https://doi.org/10.1016/j.supflu.2019.05.005

[S12] Z.L. Yu, N. Yang, L.C. Zhou, Z.Y. Ma, Y.B. Zhu et al., Bioinspired polymeric woods. Sci. Adv. **4**(8), eaat7223 (2018). https://doi.org/10.1126/sciadv.aat7223

[S13] J. Zhu, R. Xiong, F. Zhao, T. Peng, J. Hu et al., Lightweight, high-strength, and anisotropic structure composite aerogel based on hydroxyapatite nanocrystal and

chitosan with thermal insulation and flame retardant properties. ACS Sustain. Chem. Eng. **8**(1), 71-83 (2019). https://doi.org/10.1021/acssuschemeng.9b03953

[S14] J. Zhu, F. Zhao, R. Xiong, T. Peng, Y. Ma et al., Thermal insulation and flame retardancy of attapulgite reinforced gelatin-based composite aerogel with enhanced strength properties. Compos. Part A Appl. Sci. Manuf. **138**, 106040 (2020). https://doi.org/10.1016/j.compositesa.2020.106040

[S15] D.D. Ye, T. Wang, W. Liao, H. Wang, H.B. Zhao et al., Ultrahigh-temperature insulating and fire-resistant aerogels from cationic amylopectin and clay via a facile route. ACS Sustain. Chem. Eng. **7**(13), 11582-11592 (2019). https://doi.org/10.1021/acssuschemeng.9b01487

[S16] C. Liu, L. Wan, Q. Li, X. Sun, A. Natan et al., Ice-templated anisotropic flame-resistant boron nitride aerogels enhanced through surface modification and cellulose nanofibrils. ACS Appl. Polym. Mater. **3**(3), 1358-1367 (2021). https://doi.org/10.1021/acsapm.0c01219

[S17] J. Sun, Z. Wu, B. An, C. Ma, L. Xu et al., Thermal-insulating, flame-retardant and mechanically resistant aerogel based on bio-inspired tubular cellulose. Compos. Part B Eng. **220**, 108997 (2021). https://doi.org/10.1016/j.compositesb.2021.108997

[S18] H.G. Shi, H.B. Zhao, B.W. Liu, Y.Z. Wang, Multifunctional flame-retardant melamine-based hybrid foam for infrared stealth, thermal insulation, and electromagnetic interference shielding. ACS Appl. Mater. Interfaces **13**(22), 26505-26514 (2021). https://doi.org/10.1021/acsami.1c07363

[S19] T. Xue, W. Fan, X. Zhang, X. Zhao, F. Yang et al., Layered double hydroxide/graphene oxide synergistically enhanced polyimide aerogels for thermal insulation and fire-retardancy. Compos. Part B Eng. **219**, 108963 (2021). https://doi.org/10.1016/j.compositesb.2021.108963
